# AFM-IR Insights Into Cell Wall Remodeling and Protein Reorganization in *Candida auris* Versus *Candida albicans*

**DOI:** 10.1021/acsomega.5c11732

**Published:** 2026-03-06

**Authors:** Zuzanna Bednarczyk, Tamara Daniluk, Ewelina Piktel, Robert Bucki, Katarzyna Pogoda

**Affiliations:** † Institute of Nuclear Physics Polish Academy of Sciences, Radzikowskiego 152, Krakow 31-342, Poland; ‡ Department of Medical Microbiology and Nanobiomedical Engineering, 37801 Medical University of Bialystok, Mickiewicza 2C, Białystok 15-222, Poland; § Independent Laboratory of Nanomedicine, Medical University of Bialystok, Mickiewicza 2B, Białystok 15-222, Poland

## Abstract

*Candida auris* is an emerging multidrug-resistant pathogen that poses a serious threat to public health, while *Candida albicans* is a well-studied commensal yeast. Investigating the structural and biochemical basis of *C. auris* persistence and drug resistance requires approaches capable of resolving both global and local cellular features. Here, we applied Fourier-transform infrared spectroscopy in combination with atomic force microscopy-infrared spectroscopy measurements to examine fungal cells at multiple scales, from colony-level biochemical composition to nanoscale organization within single cells. Ethanol fixation was implemented to safely handle *C. auris*, and its effects were first assessed in *C. albicans*. While fixation induced measurable modifications in lipids, glucans, and protein secondary structures, cell morphology was maintained, and dehydration improved AFM-IR reproducibility by reducing topographical artifacts. This validation confirmed that fixed cells can serve as reliable models for nanoscale spectroscopic analysis of pathogenic fungi. Comparison of fixed *C. albicans* and *C. auris* revealed striking species-specific differences. *C. auris* exhibited a more robust and heterogeneous polysaccharide network, including enriched mannan and β-1,3-glucan content, higher lipid levels with longer chains, and distinctive protein secondary structure features at the nanoscale, such as increased antiparallel β-sheets. These structural characteristics likely contribute to its environmental resilience, virulence, and multidrug resistance. Overall, this study introduces a multiscale spectroscopic platform that captures both global and nanoscale biochemical features of fungal cells, providing unique insights into *C. auris* biology and offering a foundation for future studies on antifungal responses and pathogen diagnostics.

## Graphical abstract

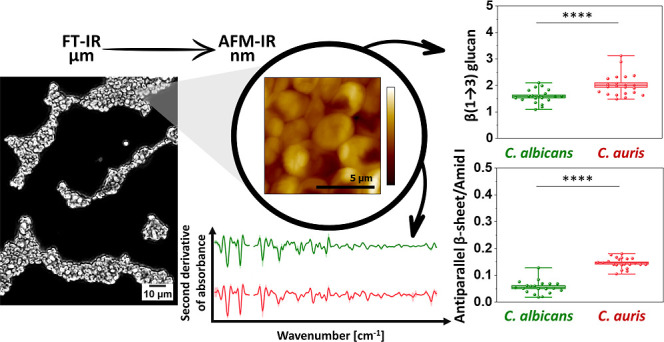

## Introduction

Fungal infections caused by *Candida* species continue to pose a major challenge, particularly in the context of antifungal resistance and persistence in healthcare environment.^1^ According to the World Health Organization Fungal Priority Pathogens List,^2^ both *Candida albicans* (*C. albicans*) and *Candidozyma auris* (formerly *Candida auris*, *C. auris*), as well as *Aspergillus fumigatus*, and *Cryptococcus neoformans*, are classified as critical priority pathogens, highlighting the urgent need for improved mechanistic understanding of their biology and drug tolerance. While *C. albicans* has long served as a model opportunistic pathogen,^3^ *C. auris* has emerged more recently as a multidrug-resistant yeast associated with persistent hospital outbreaks worldwide.^4,5^

A central determinant of fungal survival, pathogenicity, and antifungal susceptibility is the cell wall, a dynamic and multifunctional structure that provides mechanical stability, mediates adhesion and biofilm formation, and constitutes the primary interface with antifungal drugs and host immune recognition.^6^ In both *C. albicans* and *C. auris*, the cell wall follows a conserved architectural framework composed of an inner scaffold rich in chitin and β-1,3-glucans, surrounded by an outer matrix enriched in β-1,6-glucans and mannoproteins. β-1,3-glucans form the principal microfibrillar scaffold providing tensile strength and elastic support to the wall, whereas β-1,6-glucans are more highly branched and act as flexible linkers that interconnect β-1,3-glucans with other wall components, including mannoproteins, contributing to overall wall integrity. β-1,4-glucans are less common in fungal walls and are typically found in mixed-linkage glucans, where they confer distinct solubility and structural properties.^6^ Despite this shared organization, increasing evidence indicates that species-specific differences in cell wall composition and molecular organization critically influence persistence and drug resistance.^7,8^ Glycomic and proteomic analyses have demonstrated that *C. auris* cell wall mannans are highly enriched in β-1,2-linkages, which confer selective binding to human IgG and reduced recognition by innate immune receptors.^7^ These β-linked mannans contrast with the predominantly α-linked mannans in *C. albicans*, indicating that subtle carbohydrate structural differences have significant functional implications for immune evasion and colonization. Solid-state NMR and cryogenic imaging studies have recently refined our understanding of fungal cell wall nanostructure and response to antifungal stress.^8^ Moreover, the fungal cell wall is highly plastic and undergoes regulated remodeling in response to environmental and pharmacological stress.^6^ Echinocandins, which specifically inhibit β-1,3-glucan synthesis, directly target cell wall integrity, and susceptibility to these agents reflects the vulnerability of the cell wall to antifungal drugs.^6^ Notably, *C. albicans* and *C. auris* exhibit distinct adaptive responses to echinocandin exposure, indicating fundamental differences in how these species maintain wall integrity under stress.^8^ Biological strategies of *C. albicans* relies on morphological plasticity, while *C. auris* has evolved biochemical adaptations that favor persistence and resistance.^8^

Both *C. albicans* and *C. auris* are capable of forming biofilms, which contribute to persistence and antifungal resistance.^9^ Importantly, both species are capable of morphological variation: *C. auris* exhibits strain-dependent phenotypic plasticity, including the formation of pseudohyphae and aggregative multicellular forms,^10^ whereas *C. albicans* relies on yeast, pseudohyphal, and true hyphal morphologies^11^ to drive tissue invasion and immune modulation. These phenotypic differences suggest underlying nanoscale variations in morphology and cell wall architecture that remain insufficiently characterized.

Vibrational spectroscopic techniques such as Fourier-transform infrared (FT-IR) spectroscopy have been widely used to assess the global biochemical composition of fungal cells. Nevertheless, most studies to date have focused on nonpathogenic or commensal yeasts.^12–14^ What is more, conventional FT-IR provides ensemble-averaged information and lacks the spatial resolution required to resolve nanoscale chemical heterogeneity within the fungal cell wall. Atomic force microscopy-infrared spectroscopy (AFM-IR) overcomes this limitation by enabling localized infrared measurements with nanometer-scale spatial resolution, directly correlating chemical composition with morphology and mechanical properties within single fungal cells. To date, nanoscale infrared characterization of the *C. auris* cell wall has not been reported, representing a critical gap in our understanding of its structural organization.

In this study, we establish a multiscale spectroscopic framework that integrates FT-IR and AFM-IR to systematically compare the biochemical composition and morphology of *C. albicans* and *Candidozyma auris*. By correlating colony-level molecular fingerprints with nanoscale chemical organization, we identify reproducible, species-specific infrared signatures and provide the first nanoscale infrared characterization of *C. auris*. This approach establishes AFM-IR as a powerful tool for studying pathogenic fungi and provides a foundation for future investigations into cell wall remodeling, antifungal resistance, and diagnostic development.

## Results and Discussion

### From the Colony to the Cell: Spectral Characterization of Commensal *C. albicans* and Multidrug-Resistant *C. auris* by FT-IR and AFM-IR

Hyperspectral imaging based on infrared (IR) spectroscopy provides a powerful means to obtain biochemical information in two complementary dimensions: spectral fingerprints of cellular constituents and their spatial distribution within the sample, visualized as chemical maps on a micrometer scale. This dual capability allows for a more complete understanding of the molecular organization of cells and the biochemical shifts that may accompany phenotypic changes. The strength of conventional Fourier-transform infrared (FT-IR) imaging lies in its ability to rapidly collect spectra from large fields of view, thereby delivering statistically robust, population-level insights into cellular composition of colony. However, its typical spatial resolution of approximately 1.1 μm restricts the possibility of resolving alterations in molecular architecture of the fungal cell, which are often crucial for understanding the fine structural and biochemical rearrangements occurring at the subcellular level. To overcome this limitation, AFM-IR integrates the spatial precision of atomic force microscopy (AFM) with the chemical specificity of IR spectroscopy, enabling the acquisition of spectra and chemical maps with a resolution approaching approximately 30 nm (in contact mode). Thus, combining bulk FT-IR analyses with nanoscale AFM-IR measurements provides a hierarchical view of fungal organization from global biochemical profiles to local molecular rearrangements. Such a multiscale approach allows us to dissect how subtle nanoscale modifications in cell wall components, lipids, and proteins translate into broader phenotypic differences between *Candida* species. However, in order to safely perform spectroscopic measurements on *C. auris*, additional precautions were required. This species, in contrast to the commensal *C. albicans*, is characterized by its multidrug resistance, high persistence in the hospital environment, and the ability to cause severe systemic infections.^15,16^ To minimize any biological risk and ensure safe sample preparation, fungal cells were subjected to deactivation prior to spectral analysis using 99.6% ethanol rinsing.

### Impact of Ethanol Fixation on the Biochemical Profile of *C. albicans*

Different approaches of sample preparation were systematically investigated, and comparisons between them demonstrated that the preparation method significantly affects both spectral quality and the ability to discriminate among *Candida* species.^17^ In designing the experimental approach, two key conditions were prioritized: ensuring safe handling of the material and working with dried samples, as the presence of water is known to impose significant limitations in infrared spectroscopy. Residual moisture introduces strong absorption bands in the mid-infrared region that overlap with biologically relevant vibrational modes, particularly in the amide I region, and can lead to baseline distortions. Drying the samples therefore minimizes spectral interference and hydration-induced variability, resulting in improved signal-to-noise ratio, enhanced spectral reproducibility, and more reliable comparison of band intensities and positions across samples. To fulfill these requirements while preserving as much spectral information as possible, ethanol deactivation was selected as the preferred procedure. Importantly, the aim of this step was not disinfection but rapid fixation and chemical deactivation of yeast cells. Nevertheless, 70%, 80%, 90%, 99.6% ethanol concentrations were tested for rapid fixation (Figure S1 in Electronic Supporting Information). For hydrated alcohol, significant collapse of the cell structure (visible areas of subsidence, indicated by white arrows in Figure S1) and even damage to the cell wall surface were observed (in the case of 90%, see insert Figure S1). On the other hand, exposure to 99.6% ethanol generally exerts multiple effects on cells, including protein denaturation, lipid peroxidation, and disruption of membrane.^18,19^ In the yeast case, the use of 99.6% ethanol (Figure 1), characterized by a minimal water content, enabled fast dehydration and effective metabolic arrest while limiting water-induced swelling, leaching, or redistribution of intracellular components. This was particularly critical for yeast cells, whose rigid cell wall requires efficient penetration to ensure biosafety during handling, yet preservation of the native chemical composition, especially lipid and protein signatures, essential for reliable FT-IR and AFM-IR analyses. It is worth noting that after applying various fixation procedures, we did not observe any fungi growth in broth medium during 48 h incubation, which indicates effective inactivation of the cells.

**Figure 1:**
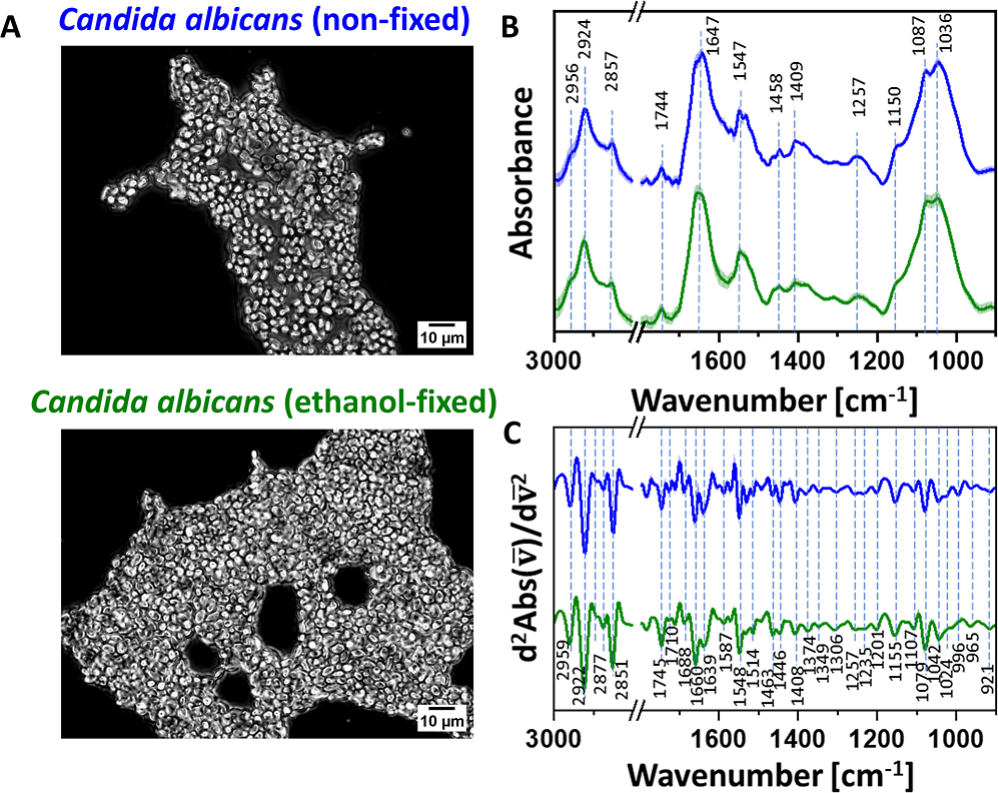
Global FT-IR characterization of *C. albicans* after ethanol fixation. Panel A: bright-field optical microscopy images of non-fixed (top) and ethanol-fixed (bottom) *Candida albicans*. Scale: 10 μm. Panel B: average FT-IR absorption spectra (solid lines) with standard deviations (shaded regions) for non-fixed and ethanol-fixed *C. albicans* (blue and green, respectively). Panel C: average second-derivative analysis of the FT-IR spectra. The bands’ assignment is given in Table 1. Enlarged versions of Panels B and C are provided in the Supporting Information, Figure S2.

Light-transmission optical microscopy of *C. albicans* (Figure 1A) following the fixation procedure did not reveal any changes at the macroscopic level. Cells maintained their typical morphology, with no visible cell wall damage or structural deformation, suggesting that the ethanol fixation effects are not observable using conventional light microscopy. FT-IR spectroscopy was employed to probe molecular-level alterations, providing complementary data at a similar micrometer-scale resolution. In complex biological samples, many absorption bands overlap and are intrinsically broad (Figure 1B), e.g., the amide I band, which contains information on protein secondary structure. To resolve these features, second derivative spectra were calculated (Figure 1C). This procedure transforms subtle inflections into distinct, sharp bands, enabling the precise identification of overlapping signals and providing detailed insight into molecular conformation and composition. Assignments of the observed bands are summarized in Table 1.

**Table 1:** IR Band Assignments for Functional Groups Found in the 2nd Derivative Spectra of *C. albicans* and *C. auris* Measured *via* FT-IR and AFM-IR^a^

FT-IR					AFM-IR		
C. albicans(non-fixed)	C. albicans(ethanol-fixed)	C. auris(ethanol-fixed)	assignment	origin	C. albicans(non-fixed)	C. albicans(ethanol-fixed)	C. auris(ethanol-fixed)
2958	2960	2959	ν_as_CH_3_ ^20^	lipids	2959	2959	2960
2922	2923	2922	ν_as_CH_2_ ^20^	lipids	2924	2924	2923
2892	2892	2892	δCH^21^	lipids, proteins	2893	2894	2892
2875	2875	2877	ν_s_CH_3_ ^20^	lipids	2875	2875	2876
2850	2852	2851	ν_s_CH_2_ ^20^	lipids	2852	2852	2852
1745	1745	1745	νC=O^20^	lipid esters/amide I	1746	1746	1746
1710	1711	1710	νC=O^20^	lipid esters/amide I	1716	1713	1711
1688	1687	1688	parallel β-sheet^22^	amide I (proteins)	1684	1682	1682
-	-	-	β-turn^23^	amide I (proteins)	-	1673	-
1660	1659	1660	α-helix^23^	amide I (proteins)	1656	1653	1656
1639	1638	1639	antiparallel β-sheet^23^	amide I (proteins)	1636	1632	1634
-	-	-	extended chains^23^	**amide I (proteins)**	-	1616	-
1587	1587	1587	νC=C^23^	tyrosine (proteins)	1593	1595	1593
1570	1571	1571	ν_as_COO^–^ ^23^	proteins	1567	1566	1566
1548	1547	1548	antiparallel β-sheet^22^	amide II (proteins)	1545	1540	1546
1514	1514	1514	tyrosine ring mode^23^	amide II (proteins)	1516	1518	1517
1464	1463	1463	δ_scissor_CH_2_ ^24^	acyl chain of lipids	1460	1460	1462
1446	1446	1446	δCH_2_ ^21^	proteins and peptides	1438	1437	1438
-	-	-	δC–O–H^21^	proteins	1410	1414	1414
1406	1408	1408	ν_s_COO^–^ ^17,21^	proteins, carboxylic acids, free amino acids	1391	1391	1389
1380	1374	1374	γCH_2_ ^17,21,25^	lipids, β-1,3 glucans	-	-	-
-	-	-	α-helix^26^	amide III (proteins)	1314	1315	1315
1304	1307	1306	α-helix^26^	amide III (proteins)	1298	1295	1286
1256	1256	1257	random coil^26^	amide III (proteins)	1259	1259	1259
1234	1234	1235	β-sheet^26^	amide III (proteins)	1246	1246	1242
1201	1203	1201	ν_as_PO_2_^–^ ^27^	phosphomannan	1202	1203	1203
1154	1155	1155	νC–O^17,25^	β-1,3-glucans	1157	1160	1157
1107	1107	1107	νC–O^17,28^	β-1,3-glucans	1110	1113	1113
1079	1078	1079	ν_s_PO_2_^–^ ^17,21,22,24,27–29^	RNA, DNA, phospholipids	1083	1083	1082
1045	1043	1042	νC–O coupled with δC–O^22,24,27^	mannans, glycogen	1048	1048	1047
1026	-	1024	νC–C_skeletal_ coupled with δCH_2_ ^17,21,22,24,27,29^	glycogen, β-1,4-glucans	1026	1028	1025
-	1011	-			-	1011	-
996	-	996	νC=C δC–O^17,21,22,27–29^	β-1,6-glucans	995	-	994
962	964	965	νC=C, νC–O, δC–O^17,22,27^	mannans, phosphodiesters, DNA	968	968	969
-	-	-	pyranose ring vibrations^30^	mannans	-	-	938
913	915	921	ν_as_ pyranose ring^21,27,29^	glucans, mannans	-	-	-
-	-	899	pyranose ring vibrations^21,30^	mannans	-	-	904
-	-	-	α-glycosidic linkage vibrations^21,30^	mannans	859	861	861
			pyranose ring vibrations^21,30,31^	mannans	804	804	802

a ν_as_ = asymmetric stretch; ν_s_ = symmetric stretch; δ_s_ = symmetric in-plane deformation (bend); δ_as_ = asymmetric in-plane deformation (bend); γ = out-of-plane deformation.

In this study, we observed that the FT-IR spectra of ethanol-fixed *C. albicans* exhibited the presence of several characteristic bands associated with polysaccharides, proteins, and lipids (Table 1). Notable differences were identified when compared to the spectra of non-fixed cells, particularly concerning specific polysaccharide-related bands. A significant observation was the absence of the band at approximately 1026 cm^–1^ in the spectra of ethanol-fixed *C. albicans*. This band is typically attributed to C–C skeletal vibrations coupled with CH_2_ deformation modes in glycogen and β-1,4-glucans. Additionally, the band at 996 cm^–1^, typically attributed to β-1,6-glucans, was also absent in the spectra of ethanol-fixed cells. Interestingly, a new band appeared at approximately 1011 cm^–1^ in the ethanol-fixed *C. albicans* spectra. This band may be associated with C=C torsional vibrations in lipids.^29^ However, given the disappearance of bands 1026 and 996 cm^–1^, it is more likely that this band is related to glucans and suggests alterations in the content, structure, or conformation upon alcohol fixation. Glycogen and β-glucans are essential components of the fungal cell wall, and their modification may indicate changes in cell wall integrity. To assess the extent of molecular alterations, a semiquantitative analysis of the relative contributions of lipids and proteins was performed (Figure 2), alongside an estimation of changes in protein secondary structure. The intensity of characteristic FT-IR bands, including those in the amide I and II regions, was used to evaluate protein content and secondary structure composition, while lipid-associated bands (high wavenumber region and 1746 cm^–1^ band) provided insight into modifications of the cellular lipid fraction.

**Figure 2:**
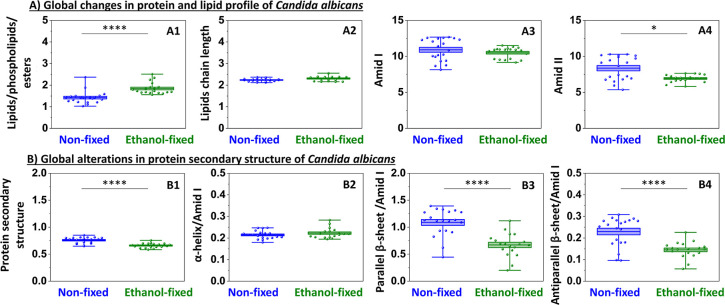
Global FT-IR analysis of *C. albicans* under ethanol fixation. Panel A: protein and lipid profile characterization: lipids/phospholipids/esters (1746 cm^–1^); lipids chain length (2960/2923 cm^–1^); amid I (1608–1698 cm^–1^); amid II (1479–1571 cm^–1^). Panel B: changes in protein secondary structure based on the second derivative of the amide I band: protein secondary structure (1479–1571 cm^–1^/1608–1698 cm^–1^); α-helix/amid I (1656 cm^–1^/1608–1698 cm^–1^); parallel β-sheet/amid I (1682 cm^–1^/1608–1698 cm^–1^); antiparallel β-sheet (1634 cm^–1^/1608–1698 cm^–1^). Values shown in box plots: mean (horizontal line), SD (box), minimal and maximal values (whiskers). Statistical significance: *p < 0.05, **p < 0.01, ***p < 0.001, ****p < 0.0001.

Our measurements demonstrated that ethanol fixation exerted the strongest global impact on lipid amounts (Figure 2A1) and protein secondary structure (amide II/amide I, Figure 2B1). An increased signal was observed at 1746 cm^–1^, corresponding to lipid esters (Figure 2A1 and Table 1). Importantly, this enhancement does not reflect lipid uptake or de novo lipid biosynthesis but rather is consistent with cell dehydration and compression of intracellular material upon ethanol fixation. It might be concluded that, because the same absolute amount of lipids becomes confined to a smaller cellular volume, the FT-IR signal intensity increases. Notably, no evidence of either elongation or shortening of lipid acyl chains (ν_as_ CH_3_/CH_2_) was observed (Figure 2A2), indicating that the chemical composition of lipids remained largely unchanged. Simultaneously, a statistically significant decrease in parallel and antiparallel β-sheets is observed (Figure 2B3,B4, respectively). In our study, the parallel β-sheet/amide I ratio is based on the 1682 cm^–1^/amide I band (Table 1). However, the literature shows considerable discrepancies regarding this assignment. Some studies attribute this band specifically to parallel β-sheets,^22,29^ aggregated β-sheets,^24^ and antiparallel β-sheets or even β-turn.^27^ This uncertainty should be considered when interpreting the mentioned ratio. The absence of random coil bands (around 1640–1648 cm^–1^), along with the unchanged α-helical content (Figure 2B2), suggests that ethanol treatment did not induce classical unfolding of proteins into disordered conformations, although a decrease in the overall intensity of the amide II band is observed (Figure 2A4), while the amide I band remains largely unchanged (Figure 2A3). Instead, these observations are indicative of possible protein destabilization, likely driven by cellular dehydration and similar to molecular crowding, which alter the spatial organization of protein domains.^32^ Overall, global changes in *C. albicans* at the micrometer scale show the simultaneous increase in lipid signals and decrease in β-sheet content. Those alterations reflect compaction within the cell rather than traditional protein denaturation. Importantly, high-resolution AFM-IR measurements allowed for a more detailed investigation of these effects at the nanometer scale, taking into account the local heterogeneity of single fungal cells (Figure 3).

**Figure 3:**
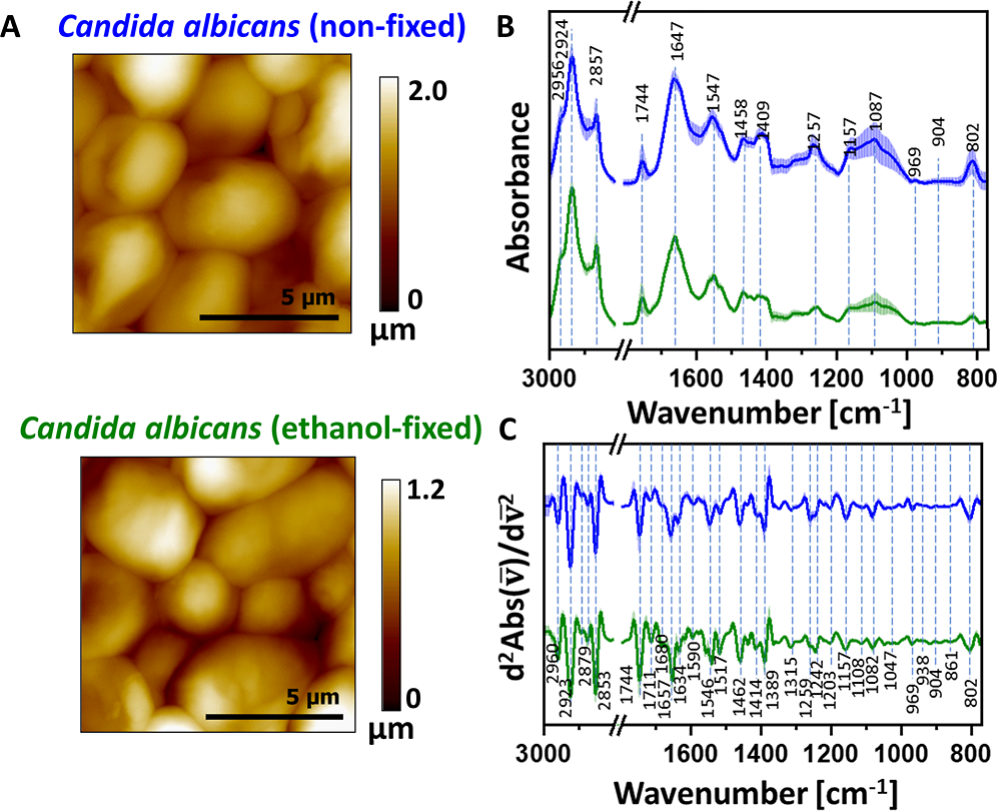
Nanoscale IR characterization of *C.albicans*. under alcohol-induced dehydration (fixation). Panel A: representative AFM topography images of non-fixed (top) and ethanol-fixed (bottom) *C. albicans*. Scale: 5 μm. Panel B: average AFM-IR absorption spectra (solid lines) with standard deviations (shaded regions) for non-fixed and ethanol-fixed *C. albicans* (blue and green, respectively). Panel C: average second-derivative analysis of the AFM-IR spectra. The bands’ assignment is given in Table 1. Enlarged versions of Panels B and C are provided in the Supporting Information, Figure S3.

Performing AFM-IR measurements on *C. albicans* cells posed considerable challenges due to their inherent size and shape. The morphology of fungal cells led to edge effects, which can generate artifacts and cause local amplification of signals, potentially distorting IR maps and affecting the interpretation of chemical composition at the nanoscale. This is particularly noticeable at cell peripheries, where tip-sample interactions are less uniform. Ethanol fixation causes dehydration and partial collapse of cells (Figure 3A), making them flatter (the height drops from a maximum of 2 to 1.2 μm), which reduces the number of artifacts and facilitates more reproducible AFM-IR measurements. Although AFM-IR spectroscopic maps were acquired for both non-fixed and ethanol-fixed cell samples, only the maps corresponding to ethanol-fixed cells were included in the manuscript. The AFM-IR maps of non-fixed cells exhibited a significant number of artifacts, primarily arising from the considerable height of the samples (up to ∼2 μm for non-fixed cells, compared to ∼1.2 μm for ethanol-fixed cells). Such pronounced height variations generate edge-related effects that manifest as artificial signal enhancement which in turn leads to a misrepresentation of the true spectroscopic response. Consequently, the resulting maps cannot be reliably interpreted in terms of chemical composition or molecular structure. In particular, cells with greater thickness and rounded morphology exacerbate edge effects, thereby reducing the accuracy of the spectroscopic data. To mitigate these challenges, further methodological optimization will be required.

Analysis of the spectral profile of *C. albicans* (Figure 3C) following ethanol fixation revealed the appearance of a band at 1673 cm^–1^ and 1616 cm^–1^, attributable to β-turn structures and side chains, respectively. Notably, these features were observed only after fixation and exclusively at the nanoscale using AFM-IR, remaining undetectable in conventional FT-IR spectra. These findings suggest that alcohol treatment induces local conformational rearrangements in proteins. The absence of these bands in conventional FT-IR can be attributed to the averaging effect, which masks subtle or spatially confined structural changes that AFM-IR can resolve owing to its nanometric spatial resolution. Furthermore, the use of a spectral resolution of 2 cm^–1^ in AFM-IR enabled the precise identification of subtle shifts in secondary structure bands. Ethanol fixation induced a shift of secondary structure bands within the amide I region toward lower wavenumbers. This shift may also reflect subtle amplification in hydrogen bonding patterns consistent with partial structural rearrangements rather than extensive unfolding. Alterations were also observed in the amide II region, corresponding to antiparallel β-sheet structures, and in the amide III region, associated with α-helices, indicating that alcohol fixation affects multiple elements of protein secondary structure. In contrast, shifts toward higher wavenumbers were detected for C–O–H vibrations in proteins and C–O vibrations in glucans, pointing to possible modifications in local bonding environments. In the literature, spectral shifts within the polysaccharide region (1200–800 cm^–1^) have been reported for *C. albicans* exposed to antifungal treatments. In particular, bands attributed to β-1,3- and β-1,6-glucans were observed to shift toward higher wavenumbers, and this trend has been associated with weakened intermolecular interactions.^33^ Importantly, the application of AFM-IR provided not only insights into localized nanoscale changes but also semiquantitative information on cell wall composition (Figure 4), which represents approximately 20% of the dry mass of a fungal cell.^34^ In particular, mannans, whose characteristic bands appear below 900 cm^–1^ and could be analyzed using AFM-IR, whereas conventional FT-IR failed to resolve these signals due to detection limitations.

**Figure 4:**
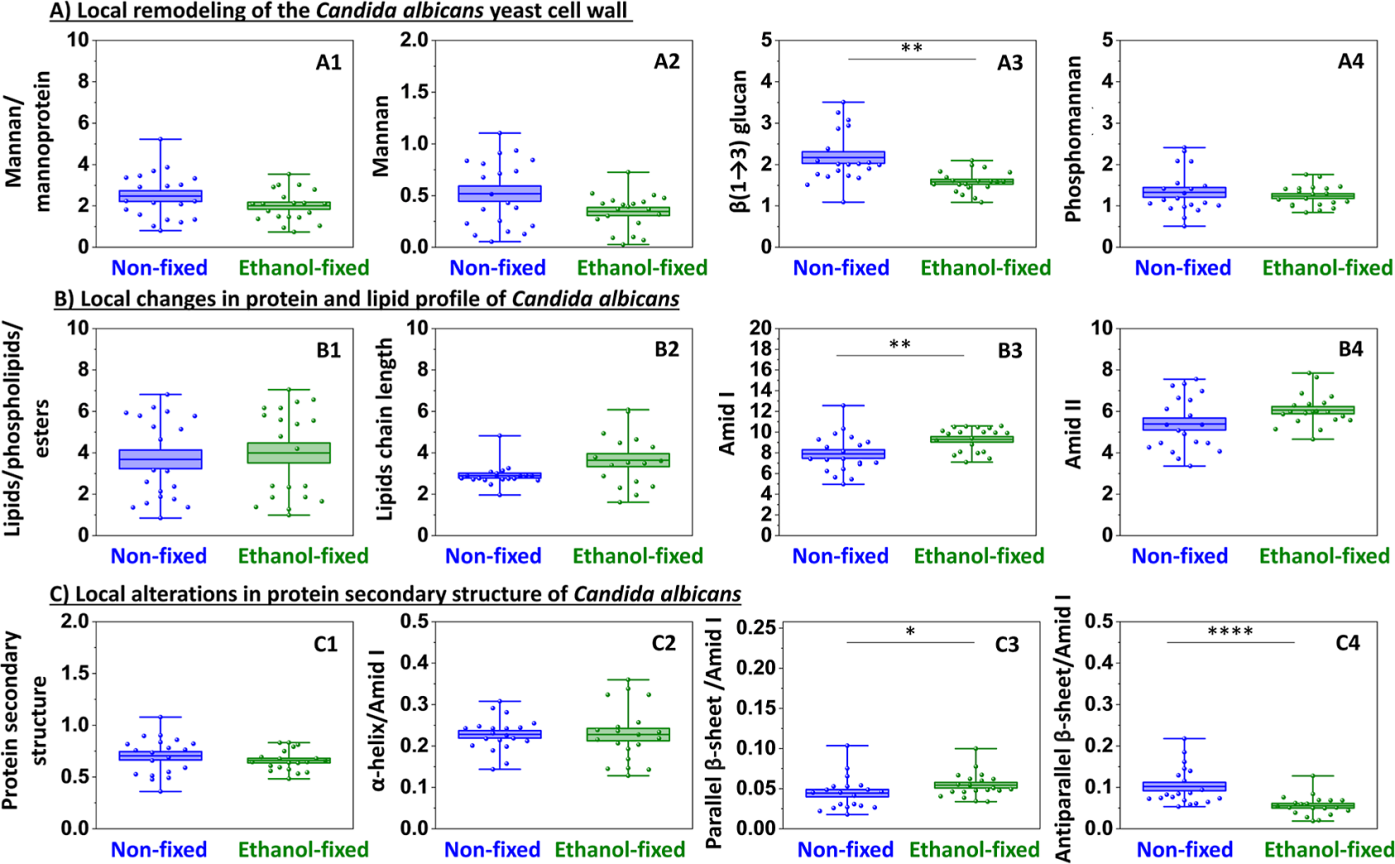
Nanoscale IR analysis of *C. albicans* under ethanol fixation. Panel A: local semiquantitative analysis of cell wall components: mannan/mannoprotein (802 cm^–1^); mannan (969 cm^–1^); β-(1,3)-glucans (1157 cm^–1^); phosphomannan (1200 cm^–1^). Panel B: protein and lipid profile characterization: lipids/phospholipids/esters (1746 cm^–1^); lipids chain length (2960/2923 cm^–1^); amid I (1608–1698 cm^–1^); amid II (1479–1571 cm^–1^). Panel C: changes in protein secondary structure based on the second derivative of the amide I band: protein secondary structure (1479–1571 cm^–1^/1608–1698 cm^–1^); α-helix/amid I (1656 cm^–1^/1608–1698 cm^–1^); parallel β-sheet/amid I (1682 cm^–1^/1608–1698 cm^–1^); antiparallel β-sheet (1634 cm^–1^/1608–1698 cm^–1^). Values shown in box plots: mean (horizontal line), SD (box), minimal and maximal values (whiskers). Statistical significance: *p < 0.05, **p < 0.01, ***p < 0.001, ****p < 0.0001.

Ethanol fixation leads to a decreasing trend in the major polysaccharide components (Figure 4A1–A4). The *C. albicans*’ cell wall is predominantly composed of microfibrillar polymers,^35^ including β-glucans (47–60% of the cell wall’s total mass) and chitin (0.6–9%), along with other polysaccharides such as mannans—polymers of mannose constituting approximately 40% of the total cell wall polysaccharide content.^36^ Among these, a statistically significant reduction was detected only for β-1,3-glucans (Figure 4A3). Since these glucans constitute the inner structural and immune scaffold of the wall,^37^ their selective decrease suggests that alcohol fixation may compromise the cell’s protective barrier. This selective decrease in cell wall components demonstrates that alcohol fixation does not remove or completely degrade polysaccharide structures, especially since the significant change is visible in the components of the inner part of the cell wall, not the outer one. What is more, AFM-IR and FT-IR produce a divergent trend following alcohol fixation of *Candida* cells. In AFM-IR spectra, we observed an increase in the amide I band (Figure 4B3), while the amide II band remains largely unchanged (Figure 4B4) as well as a decrease in antiparallel β-sheets (Figure 4C4) together with an increase in parallel β-sheets (Figure 4C3), and the appearance of a β-turn band (Table 1). By contrast, FT-IR spectra showed no evidence of β-turns and instead demonstrated decreases in amide II (Figure 2A4) as well as in both parallel and antiparallel β-sheet components (Figure 2B3,B4). The contrasting trends observed in protein-associated bands between AFM-IR and FT-IR can be interpreted in the context of: 1/heterogeneous reorganization of proteins and 2/the different spatial scales of the two techniques, and 3/the different phenomena they rely on (thermal expansion induced by IR absorption versus direct IR absorption by molecular vibrations, respectively). AFM-IR probes local nanometer-sized domains, where alcohol-induced dehydration and partial collapse of the cell can cause local compaction of proteins. In these confined regions, parallel β-sheets and β-turn motifs become more prominent, leading to the increases detected by AFM-IR. At the same time, more fragile or solvent-exposed antiparallel β-sheets are disrupted, explaining their decrease. FT-IR, on the other hand, averages the signal over the entire colony. In this ensemble view, the local increases are masked by a general loss of protein signal due to dehydration, fixation and partial structural disruption, resulting in an overall decrease of amide II and β-sheet bands. This suggests that alcohol fixation does not uniformly degrade proteins, but instead causes local compaction and global destabilization caused by cellular dehydration and similar to molecular crowding.^32^ No changes were detected in the intensity of the lipid-related band or in the lipid chain length indicator (Figure 3B1,B2) as well as protein secondary structure or α-helix/Amid I (Figure 3C1,C2). Moreover, the complementary information from both nano- and microscale methods provides a more complete picture of the fungal cell and colony. Thus, the use of ethanol fixation created a reliable experimental platform to investigate both global and local alterations between commensal and multidrug-resistant *Candida* species (Figure 5), while maintaining structural and chemical features.

**Figure 5:**
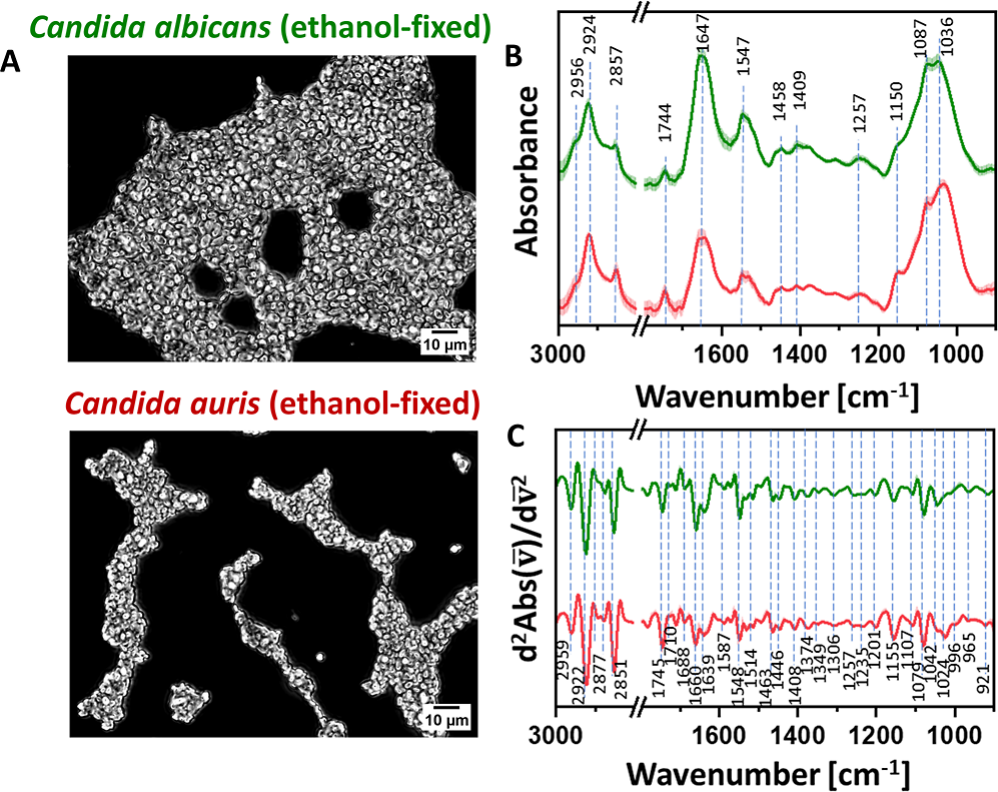
Global FT-IR characterization of *C. albicans* and *C.auris*. Panel A: bright-field optical microscopy images of *C. albicans* (top) and *C. auris* (bottom). Scale: 10 μm. Panel B: average FT-IR absorption spectra (solid lines) with standard deviations (shaded regions) for *C. albicans* and *C. auris* (green and red, respectively). Panel C: average second-derivative analysis of the FT-IR spectra. The bands’ assignment is given in Table 1. Enlarged versions of Panels B and C are provided in the Supporting Information, Figure S4.

### Comparative Analysis of the Biochemical Differences between *C. albicans* and *C. auris*

Under light microscopy, *C. albicans* and *C. auris* have a similar size and oval to ellipsoidal shape of cells (Figure 5A) with a size of around 5–6 μm^38^ and 2.5–5 μm,^39^ respectively. Although both species share the canonical fungal cell–wall skeleton, an inner layer of chitin and β-1,3/β-1,6-glucans with an outer mannoprotein-rich fibrillar coat, high-impact techniques have revealed critical species-specific differences.^40^ The overall spectral profile of both *Candida* species appeared similar (Figure 5B,C). However, two notable differences were observed. In *C. auris*, the same band arrangement was detected as in metabolically active (non-fixed) *C. albicans*. Specifically, characteristic absorption bands at 1024 and 996 cm^–1^, attributed to glucans, were present, while the band at 1011 cm^–1^ was absent. This spectral pattern suggests that exposure to alcohol may not have altered the cell wall architecture of *C. auris* as profoundly as it did in *C. albicans*. Those observations could therefore indicate a greater structural resilience of *C. auris* cell wall under alcohol treatment. Interestingly, in the spectra of *C. auris*, an additional absorption band was detected at 899 cm^–1^, which was not observed in either non-fixed and ethanol-fixed cells of *C. albicans.* This band is typically associated with mannans, key polysaccharides present in the outer layer of the fungal cell wall.^30^ The appearance of this feature exclusively in *C. auris* may indicate a distinct organization or increased surface exposure of mannan structures in this species. According to the literature, the mannan of *C. auris* is distinct from mannans of other pathogenic *Candida* species, primarily due to its high content of β-1,2 linkages.^7^ This observation may be a clue to the importance of conformations or bonds in mannans in *C. auris* that may contribute to its survival in hostile environments and its clinical relevance as an emerging pathogen. Nevertheless, an in-depth statistical analysis of the integral intensities of individual bands (Figure 6) showed the multifaceted differences between *C. auris* and *C. albicans*.

**Figure 6:**
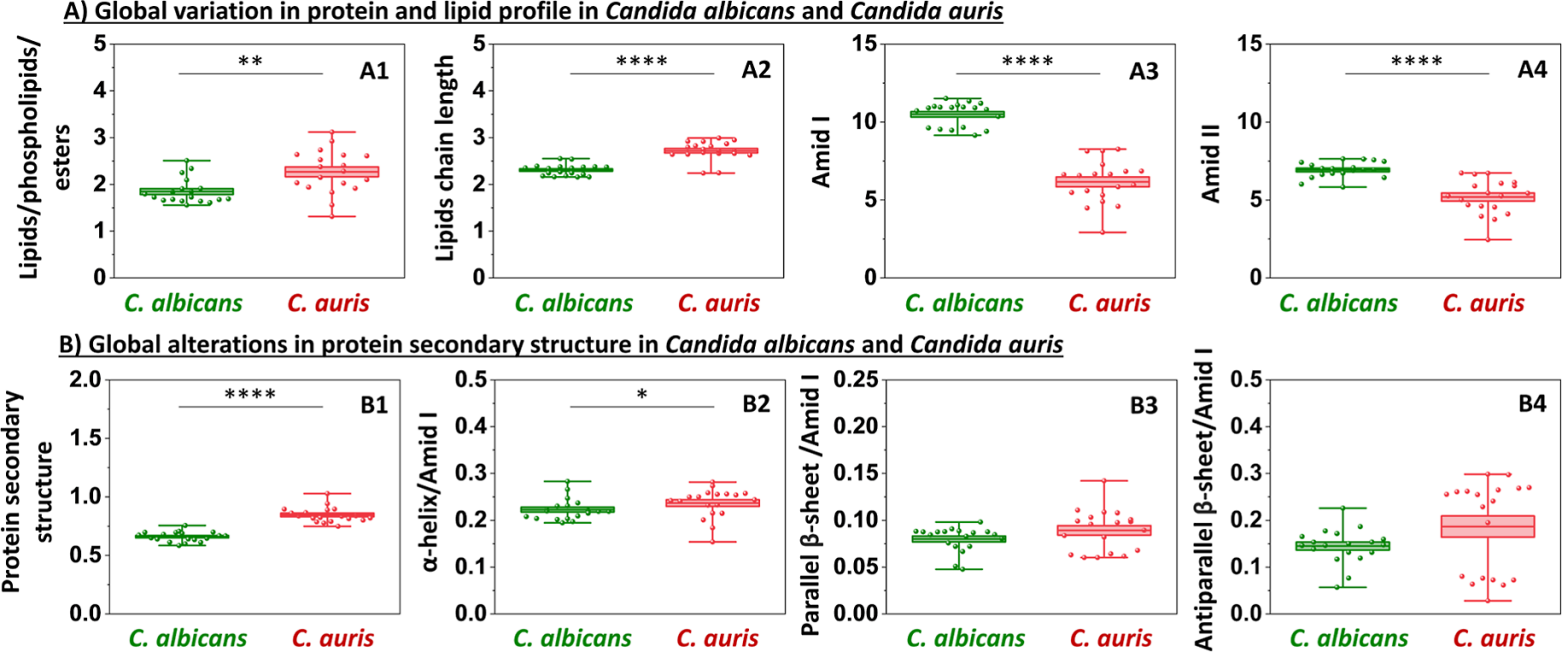
Global insights into the chemical profile of persistence and antifungal resistance in *C. auris*­(red)­versus *C. albicans*­(green). Panel A: protein and lipid profile characterization: lipids/phospholipids/esters (1746 cm^–1^); lipids chain length (2960/2923 cm^–1^); amid I (1608–1698 cm^–1^); amid II (1479–1571 cm^–1^). Panel B: changes in protein secondary structure based on the second derivative of the amide I band: protein secondary structure (1479–1571 cm^–1^/1608–1698 cm^–1^); α-helix/amid I (1656 cm^–1^/1608–1698 cm^–1^); parallel β-sheet/amid I (1682 cm^–1^/1608–1698 cm^–1^); antiparallel β-sheet (1634 cm^–1^/1608–1698 cm^–1^). Values shown in box plots: mean (horizontal line), SD (box), minimal and maximal values (whiskers). Statistical significance: *p < 0.05, **p < 0.01, ***p < 0.001, ****p < 0.0001.

Both the protein–lipid profile (Figure 6A) and the secondary structure of proteins (Figure 6B) differ markedly between the two *Candida* spp. *C. auris* is characterized by a higher lipid content as well as longer lipid chain lengths compared to *C. albicans* (Figure 6A1,A2). It has already been confirmed that lipids play a critical role in fungal stress resistance and antifungal drug tolerance.^4,41^ The enrichment of longer-chain lipids in *C. auris* may contribute to a more rigid and less permeable cell membrane, thereby enhancing resistance to environmental stressors, including alcohol exposure and antifungal agents. Moreover, modifications in lipid composition could influence the organization of membrane-associated proteins and, consequently, the secondary structure of cell wall proteins. A global assessment of secondary structural changes revealed a higher proportion of α-helix structures (Figure 6B2) in *C. auris.* Since α-helices are generally associated with protein stability and resistance to denaturation, this feature may reflect an adaptive strategy to preserve the functionality of membrane-associated and cell wall proteins under environmental stress. A greater α-helical content could also contribute to more compact protein folding, potentially enhancing the robustness of cell wall components.

Local analysis enabled the capture of colony population heterogeneity in *Candida* spp. AFM images revealed that *C. auris* cells are smaller than *C. albicans* cells (Figure 7A). Collapsing caused by ethanol fixation reduced topographical heterogeneity, enabling the tip to maintain more consistent contact and produce interpretable nanoscale spectra. Nevertheless, edge effects remain observable in AFM-IR measurements (Figure 7A). For instance, the IR map corresponding to the 1746 cm^–1^ band, which reflects the distribution of lipids, showed apparent signal enhancement at the periphery of one of the *C. albicans* cells that represents a tip-sample interaction artifact commonly associated with edge geometries. The second distribution map was collected for the band at 802 cm^–1^, which originates from vibrational modes characteristic of mannans covalently linked to proteins in the form of mannoproteins. These mannoproteins not only contribute to the structural framework of the wall but also play key roles in its biochemical and functional properties.^36^ Interestingly, *C. auris* cells exhibited fewer artifacts compared to *C. albicans*, which may be due to greater compression of the cell material during dehydration. Furthermore, AFM-IR maps highlighted the heterogeneity of *Candida* cells, as lipid- and mannan-associated bands were not present at uniform intensities across all cells. These observations suggest that fungal cells, especially *C. auris*, exhibit both structural and chemical variability at the single-cell level, reflecting differences in cell wall composition and possibly adaptive responses to environmental stress, as well as the importance of using AFM-IR for the local biochemical characterization of fungal cells. By combining high-resolution imaging with chemical specificity, AFM-IR allowed the detection of heterogeneity in cell wall composition and molecular organization that would be otherwise masked in bulk analyses. Spectral analysis also revealed certain local differences between the examined those *Candida* spp. (Figure 7C). The band corresponding to secondary structure elements in the amide I region (α-helix) and amide II region (antiparallel β-sheet) was shifted toward higher wavenumbers in *C. auris* compared to *C. albicans*, whereas the α-helix and β-sheet bands in amide III were shifted toward lower wavenumbers. These spectral shifts suggest subtle alterations in hydrogen bonding and protein backbone conformation of *C. auris*. The absence of bands at 1673 cm^–1^ and 1616 cm^–1^, attributable to β-turn structures and side chains, suggests that alcohol exposure may not have altered the proteins’ architecture of *C. auris* as profoundly as it did in *C. albicans*. What is more, glucan-associated bands at 1157 and 1025 cm^–1^ in *C. auris* were shifted toward lower wavenumbers compared to ethanol-fixed *C. albicans*, and appear at positions similar to those observed in metabolically active (non-fixed) *C. albicans*. Moreover, the pattern of β-glucan bands in *C. auris* was also preserved. The bands at 1025 and 994 cm^–1^ were present, while the band at 1011 cm^–1^ was absent, mirroring the pattern observed in metabolically active *C. albicans* but differing from the inactive (ethanol-fixed) one. This observation further supports the notion that alcohol-induced deactivation did not significantly affect the cell wall of *C. auris*, in contrast to *C. albicans*. The structural integrity and functionality of *C. auris* cell wall remain largely preserved despite exposure to alcohol. Interestingly, in the spectra of *C. auris*, additional absorption bands were detected not only at 899 cm^–1^ but also at 938 cm^–1^. In both cases, these bands are attributed to mannans and likely reflect vibrations of additional mannan structures or different cross-linking within the cell wall. The presence of these bands, which are absent in both non-fixed and ethanol-fixed *C. albicans*, may indicate unique structural features of *C. auris* mannans associated with its multidrug-resistant phenotype.

**Figure 7:**
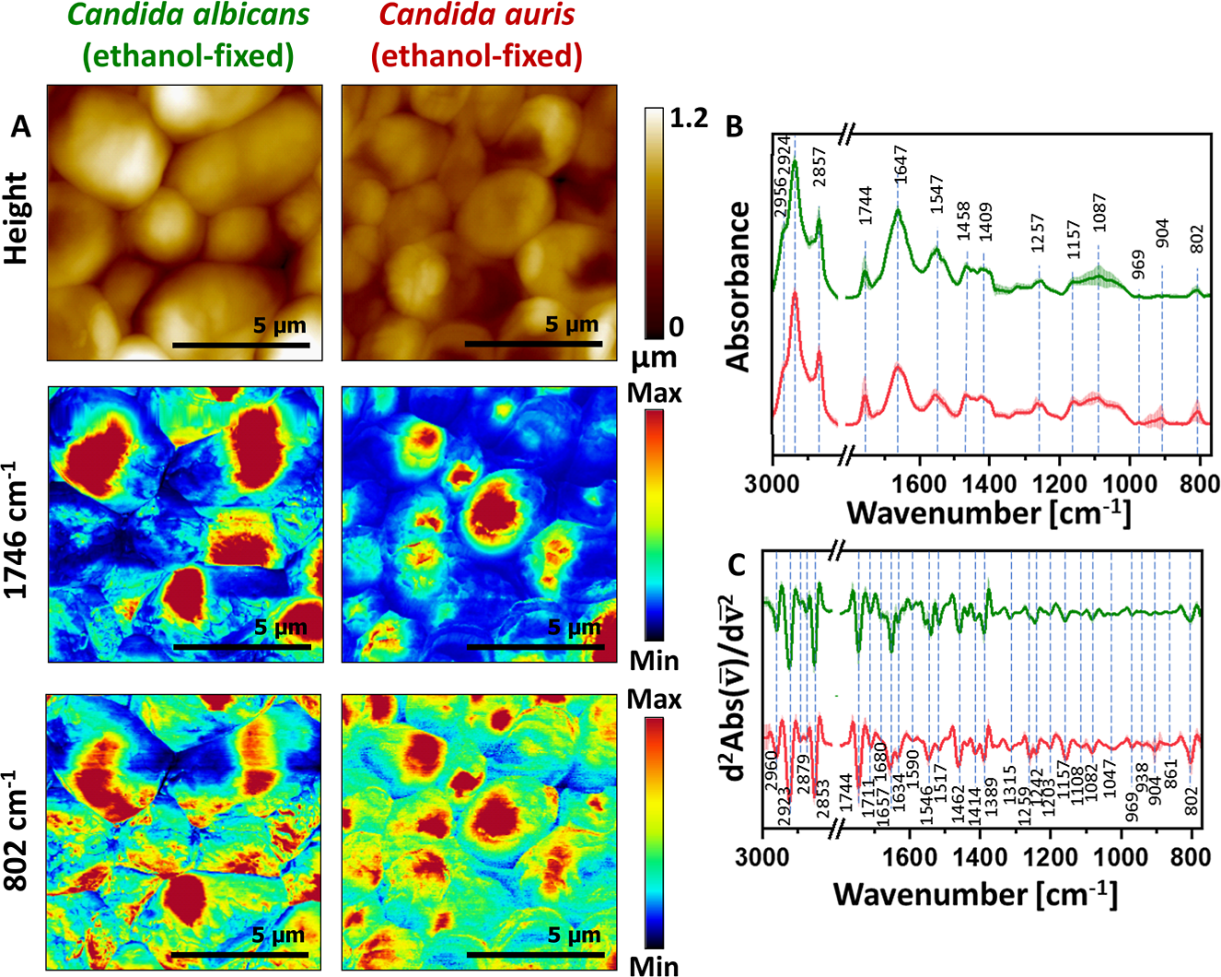
Nanoscale IR characterization of *C. albicans* and *C. auris*. Panel A: representative AFM topography images (top) and corresponding AFM-IR absorption maps recorded at 1746 cm^–1^ (middle) and 802 cm^–1^ (bottom). Scale: 5 μm. Panel B: average AFM-IR absorption spectra (solid lines) with standard deviations (shaded regions) for *C. albicans* and *C. auris* (green and red, respectively). Panel C: average second-derivative analysis of the AFM-IR spectra. The bands’ assignment is given in Table 1. Enlarged versions of Panels B and C are provided in the Supporting Information, Figure S5.

The analysis of local changes between the two *Candida* strains revealed alterations that were not detectable in global measurements, particularly in the secondary structure of proteins (Figure 8C, except α-helix/amid I in Figure 8C2). Additionally, this approach allowed for a semiquantitative assessment of the cell wall composition (Figure 8A). A statistically significant higher content of mannans and mannoproteins (Figure 8A1) was observed in the cell wall of *C. auris*, as indicated by the band at 802 cm^–1^, whereas no significant difference was detected at 969 cm^–1^ (Figure 8A2, although this band may also include contributions from DNA and other cellular components (Table 1)). Additionally, *C. auris* displayed a higher content of β-1,3-glucans (Figure 8A3) compared to *C. albicans.* No changes were detected in the intensity of the phosphomannan (Figure 8A4). These results suggest that *C. auris* has a more complex and heterogeneous cell wall polysaccharide network, which may support adaptive remodeling in response to environmental stressors. At the local level, no significant differences in lipid content (Figure 8B1,B2) were observed, and the semiquantitative assessment of amide I and II regions (Figure 8B3,B4) reflected the global protein composition. Interestingly, AFM-IR analysis revealed pronounced differences (****p < 0.0001) in the contribution of secondary structures (Figure 8C3,C4). Proteins in *C. auris* exhibited a higher proportion of antiparallel β-sheets and a lower proportion of parallel β-sheets compared to *C. albicans.* This pattern may indicate a strategic reorganization of protein secondary structures in *C. auris*, where increased antiparallel β-sheet content could enhance intermolecular interactions and local rigidity, supporting a more resilient cell wall matrix. Collectively, these findings highlight that *C. auris* exhibits a distinct structural protein–polysaccharide configuration that likely contributes to its persistence, virulence, and multidrug resistance.

**Figure 8:**
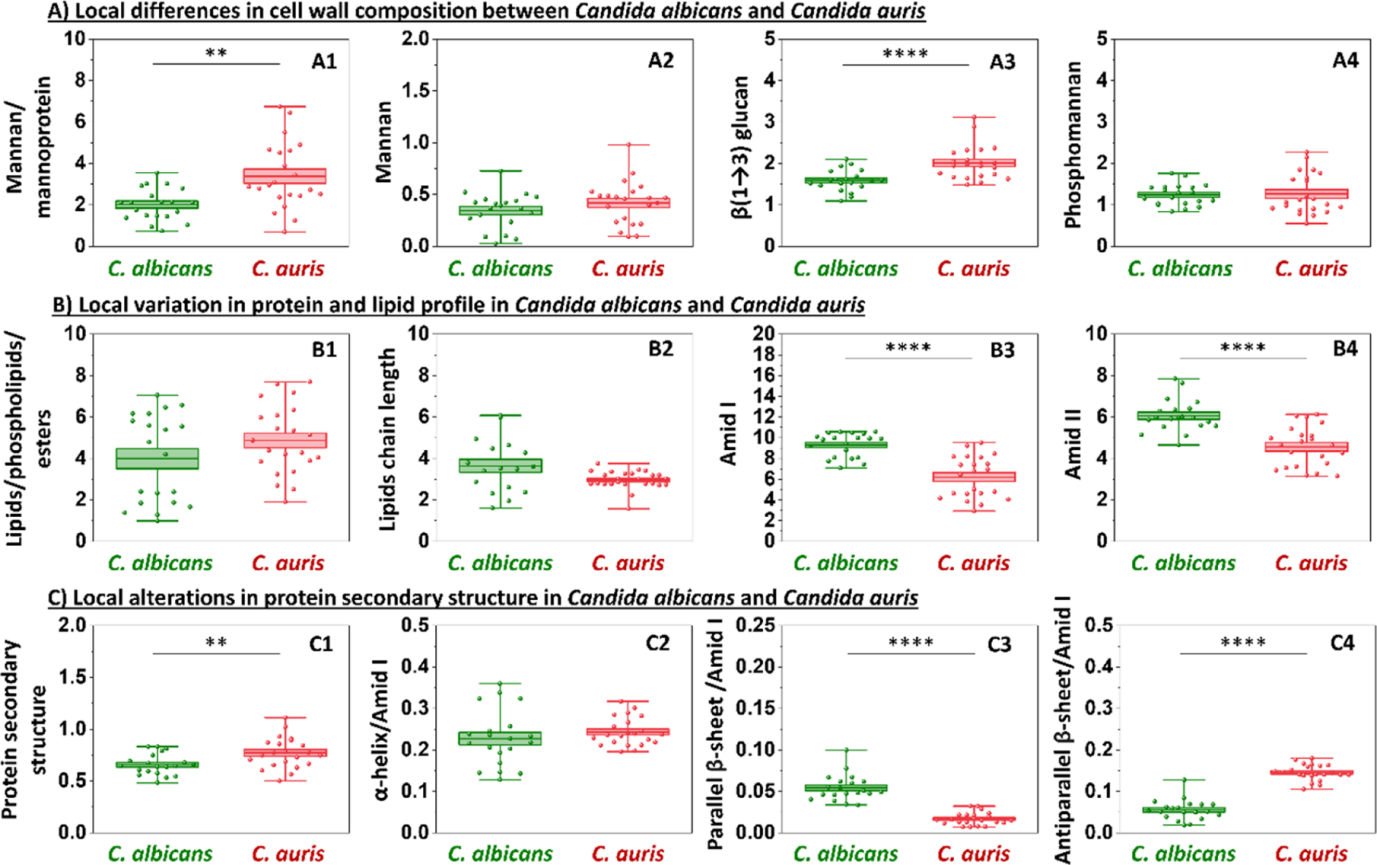
Nanoscale insights into the chemical profile of persistence and antifungal resistance in *C. auris*­(red) versus *C.albicans*­(green). Panel A: local semiquantitative analysis of cell wall components: mannan/mannoprotein (802 cm^–1^); mannan (969 cm^–1^); β-(1,3)-glucans (1157 cm^–1^); phosphomannan (1200 cm^–1^). Panel B: protein and lipid profile characterization: lipids/phospholipids/esters (1746 cm^–1^); lipids chain length (2960/2923 cm^–1^); amid I (1608–1698 cm^–1^); amid II (1479–1571 cm^–1^). Panel C: changes in protein secondary structure based on the second derivative of the amide I band: protein secondary structure (1479–1571 cm^–1^/1608–1698 cm^–1^); α-helix/amid I (1656 cm^–1^/1608–1698 cm^–1^); parallel β-sheet/amid I (1682 cm^–1^/1608–1698 cm^–1^); antiparallel β-sheet (1634 cm^–1^/1608–1698 cm^–1^). Values shown in box plots: mean (horizontal line), SD (box), minimal and maximal values (whiskers). Statistical significance: *p < 0.05, **p < 0.01, ***p < 0.001, ****p < 0.0001.

## Conclusions

This work establishes a multiscale spectroscopic framework integrating FT-IR and AFM-IR for comprehensive structural and biochemical characterization of fungal cells. By correlating colony-level molecular fingerprints with nanoscale chemical organization, this approach provides insights inaccessible to either technique alone. A critical element of the framework is the optimized sample preparation strategy, which ensures biosafety while preserving chemically relevant information. Ethanol-based rapid fixation was shown to be compatible with FT-IR and AFM-IR measurements, maintaining cellular morphology and improving AFM-IR mapping through reduced topographic artifacts. Using this methodology, we report the first nanoscale infrared characterization of *C. auris*. Importantly, the IR-based readout provides a holistic, category-specific biochemical fingerprint reflecting collective changes in major macromolecular classes rather than pinpointed identification of individual biomolecules. Within this framework, reproducible and discriminatory spectral patterns were identified, enabling reliable differentiation between *C. auris* and *C. albicans*. These species-specific differences in cell wall architecture and biochemical organization offer molecular insight into the enhanced persistence and drug resistance of *C. auris*. *C. auris* exhibited a more complex and resilient polysaccharide network, enriched mannan and β-1,3-glucan content, higher lipid levels and longer chains (observed specifically in the bulk FT-IR analysis), lower protein content, and a distinctive secondary structure profile with increased antiparallel β-sheets (observed specifically in the local AFM-IR analysis). Overall, the proposed FT-IR/AFM-IR framework represents a robust platform for fungal research and may serve as a foundation for future integration with targeted biochemical or molecular assays, enabling advanced diagnostic strategies and mechanistic studies of antifungal responses.

## Experimental Section

### Fungal Culture and Passaging

*C. auris* 21092 was obtained from DSMZ (Braunschweig, Germany). This strain was first isolated in Japan (clade II). *C. albicans* ATCC 26790 was American Type Culture Collection (ATCC). The two species were selected to represent yeasts with distinct ecological niches and epidemiological strategies, enabling comparative analysis of chemical composition. All strains were stored in glycerol solutions at −80 °C. When necessary, strains were inoculated onto Sabouraud Dextrose with chloramphenicol LAB-AGAR medium (Biomaxima, Lublin, Poland) and routinely cultured at 30 °C.

### Sample Preparation for FT-IR and AFM-IR

Yeast cell numbers were estimated based on optical density (OD). Before samples’ application, exponentially growing *Candida* cells were suspended in sterile water and diluted to the OD600 ∼0.5, which corresponded to ∼5 × 10^7^ CFU/mL. Cells were collected at mid logarithmic growth phase to ensure physiological homogeneity and maximize reproducibility of the infrared spectroscopic readout. Sampling at mid log phase minimizes variability arising from growth stage-dependent adaptive responses and allows the observed spectral differences to be attributed primarily to species-specific cell wall architecture rather than to differences in physiological state. The CaF_2_ optical slides were thoroughly washed with 70% ethanol, rinsed three times with sterile water, and exposed to UV light for 15 min before sample application. A 100 μL of the previously prepared fungal suspension in sterile water was applied to the thoroughly dried slides and left to dry at 37 °C. Rapid fixation of yeast cells was evaluated using ethanol at different concentrations (70%, 80%, 90%, and 99.6%). Cells deposited on CaF_2_ slides were immersed in ethanol for 3 s. Following fixation, samples were assessed for effective fungal deactivation by inoculation into liquid broth medium and incubation for 48 h. The absence of fungal growth during incubation confirmed successful inactivation. For future ethanol-fixed conditions, the dried samples on CaF_2_ slides were immersed in 96.6% ethanol for 3 s and then rinsed with sterile water. The procedure was repeated three times before being left to dry.

### FT-IR Measurement

FT-IR measurements were performed using a HYPERION 3000 FT-IR microscope equipped with a 36× objective and coupled to a Vertex 70v spectrometer (Bruker, Ettlingen, Germany) in transmission mode. Hyperspectral images (70 × 70 μm) were acquired with a 64 × 64 pixels (4096 individual spectra) FPA detector, yielding a projected pixel size of 1.1 μm × 1.1 μm. Hyperspectral data were acquired as matrices from 20 different regions of cells deposited on CaF_2_ optical windows (Crystran Ltd.). Spectra were acquired over the range 800–3800 cm^–1^ with a spectral resolution of 4 cm^–1^, averaging 256 scans per spectrum.

### AFM-IR Measurement

AFM-IR measurements were conducted in contact mode using a NanoIR2 spectrometer (Anasys Instruments, Santa Barbara, CA, USA). All measurements employed silicon gold-coated PR-EX-nIR2 probes with a tip diameter of 30 nm and a resonance frequency of 13 ± 4 kHz (Anasys Instruments, USA). Contact resonances were identified using an approximate 180 kHz search location combined with a 50 kHz half-width Gaussian filter. Infrared excitation was provided by a multichip tunable quantum cascade laser (QCL; MIRcat-QT, daylight solutions) operating in the 3000–2700 cm^–1^ and 1800–750 cm^–1^ ranges with a spectral resolution of 2 cm^–1^. Spectra were acquired using 16.03% laser power and 90° polarization from seven randomly selected points on each of 20 fungal cells per condition and coaveraging 256 excitation pulses. AFM topography and IR images were recorded with a cantilever scan rate of approximately 0.4 Hz over a 10 × 10 μm area with 500 × 500 measurement points. IR maps corresponding to topography images were collected at 804 cm^–1^ and 1746 cm^–1^ using 20.26% laser power, with four maps acquired per experimental group.

### Data Preprocessing

FT-IR and AFM-IR spectral data were subjected to systematic preprocessing prior to analysis. FT-IR spectra were processed using CytoSpec and OPUS (ver. 7.5, Bruker Optics, Germany), applying noise reduction followed by hierarchical cluster analysis (HCA) to isolate spectra originating from fungal cells. The resulting spectra were subsequently averaged, given baseline correction, and smoothed (rubber: 7, iteration: 7, smoothing points: 13). AFM-IR spectra were processed in analysis studio (ver. 3.14) using Savitzky–Golay smoothing (third-order polynomial, 5-point window), followed by min–max normalization. Spectra were then averaged for individual cells. FT-IR and AFM-IR spectra were converted to second-derivative form to enhance spectral features. AFM topography and AFM-IR images were processed using MountainsMap software (ver. 7.3, Digital Surf, France), applying plane correction and flattening to remove background tilt and scanner artifacts. All preprocessing steps were applied consistently across experimental groups to ensure comparability of the resulting spectral and imaging data.

### Spectral Analysis

Semiquantitative analysis of the infrared spectra was performed using OPUS (ver. 7.5, Bruker Optics, Germany) by calculating the integrated intensities of selected spectral bands. The following bands were analyzed to assess specific biochemical components: 802 cm^–1^ (mannans/mannoproteins), 969 cm^–1^ (mannans), 1157 cm^–1^ (β-(1,3)-glucans), 1200 cm^–1^ (phosphomannans), 1746 cm^–1^ (lipids, phospholipids, esters), 2960/2923 cm^–1^ (lipid chain lengths), 1608–1698 cm^–1^ (amide I), 1479–1571 cm^–1^ (amide II). Ratios of selected bands were used to assess protein secondary structure, including amide II/amide I (general changes of protein secondary structure), 1656/amide I (α-helix), 1682/amide I (parallel β-sheet), and 1634/amide I (antiparallel β-sheet). Integrated band intensities were calculated after baseline correction and normalization to allow for comparison between experimental groups. All analyses were performed consistently across FT-IR and AFM-IR data sets to ensure reliable semiquantitative assessment of the biochemical composition of individual fungal cells.

### Statistical Analysis

Statistical analyses were performed using OriginPro software. Data distribution normality was assessed with the Shapiro–Wilk test. For pairwise comparisons: if both groups passed the normality test and variances were equal, a Student’s t-test was applied; if normality was not satisfied, the nonparametric Mann–Whitney U test (also known as the Wilcoxon rank-sum test) was used. Significance levels are reported using the following notation: *p < 0.05, **p < 0.01, ***p < 0.001, ****p < 0.0001. Values were shown in box plots: mean (horizontal line), standard deviation (box), minimal and maximal values (whiskers).

## Supplementary Material



## Data Availability

The raw FT-IR and AFM-IR spectra supporting the findings of this study are publicly available in the RODBUK repository (DOI: 10.48733/IFJPAN/RNCELU). All data directly supporting the conclusions of this work are provided in the Article and in the Supporting Information.

## Notes

AFM-IR analysis of *C. albicans* under ethanol fixation at different concentrations, including deflection, lateral deflection and height images (Figure S1); global FT-IR characterization of *C. albicans* after ethanol fixation, average spectra and second-derivative analysis (Figure S2); nanoscale IR characterization of *C. albicans* after ethanol fixation, average AFM-IR spectra and second-derivative analysis (Figure S3); global FT-IR comparison of *C. albicans* and *C. auris*, average spectra and second-derivative analysis (Figure S4); nanoscale IR comparison of *C. albicans* and *C. auris*, average AFM-IR spectra and second-derivative analysis (Figure S5) (PDF)
