# Supplementary material for: AFM-IR Insights Into Cell Wall Remodeling and Protein Reorganization in Candida auris Versus Candida albicans
Source: ACS Omega. 2026 Mar 6;11(11):17720–31. doi: 10.1021/acsomega.5c11732 (PMC13019247; doi:10.1021/acsomega.5c11732)
Supplement: Supplementary file 1 [file ao5c11732_si_001.pdf]

AFM-IR insights into cell wall remodeling and protein reorganization in *Candida auris* versus *Candida albicans*

Zuzanna Bednarczyk[1], Tamara Daniluk[2], Ewelina Piktel[3], Robert Bucki[2,3], Katarzyna Pogoda[1,*]

[1]Institute of Nuclear Physics Polish Academy of Sciences, Radzikowskiego 152, 31-342 Krakow, Poland
[2] Department of Medical Microbiology and Nanobiomedical Engineering, Medical University of Bialystok, Mickiewicza 2C 15-222 Białystok, Poland
[3] Independent Laboratory of Nanomedicine, Medical University of Bialystok, Mickiewicza 2B 15-222 Białystok, Poland
* Correspondence author: katarzyna.pogoda@ifj.edu.pl

Keywords: *Candida albicans*, *Candida auris*, Atomic Force Microscopy - Infrared Spectroscopy, Fourier-Transform Infrared Spectroscopy

Content

Figure S1. AFM-IR analysis of *Candida albicans* under ethanol fixation at different concentrations. Panel A: Deflection: vertical cantilever bending signal (with setpoint offset), providing enhanced edge contrast. Panel B: Lateral Deflection (torsional/sideways response) characterization: reveals frictional and lateral mechanical properties related to cell wall composition and structural heterogeneity under different ethanol fixation concentrations. Panel C: Height: cell surface topography. Visible areas of subsidence are indicated by white arrows; local damage to the cell wall surface is shown in the inset (90% ethanol). Scale: 5 µm. Ethanol concentrations: 70%, 80%, 90%, 99.6%.

Figure S2. Global FT-IR characterization of *C. albicans* after ethanol-fixation. Panel A: Average FT-IR absorption spectra (solid lines) with standard deviations (shaded regions) for non-fixed and ethanol-fixed *C. albicans* (blue and green, respectively). Panel B: Average second-derivative analysis of the FT-IR spectra.

Figure S3. Nanoscale IR characterization of *C. albicans* after ethanol fixation. Panel A: Average AFM-IR absorption spectra (solid lines) with standard deviations (shaded regions) for non-fixed and ethanol-fixed *C. albicans* (blue and green, respectively). Panel B: Average second-derivative analysis of the AFM-IR spectra.

Figure S4. Global FT-IR characterization of *C. albicans* and *C. auris*. Panel A: Average FT-IR absorption spectra (solid lines) with standard deviations (shaded regions) for *C. albicans* and *C. auris* (green and red, respectively). Panel B: Average second-derivative analysis of the FT-IR spectra.

Figure S5. Nanoscale IR characterization of *C. albicans* and *C. auris*. Panel A: Average AFM-IR absorption spectra (solid lines) with standard deviations (shaded regions) for *C. albicans* and *C. auris* (green and red, respectively). Panel B: Average second-derivative analysis of the AFM-IR spectra.

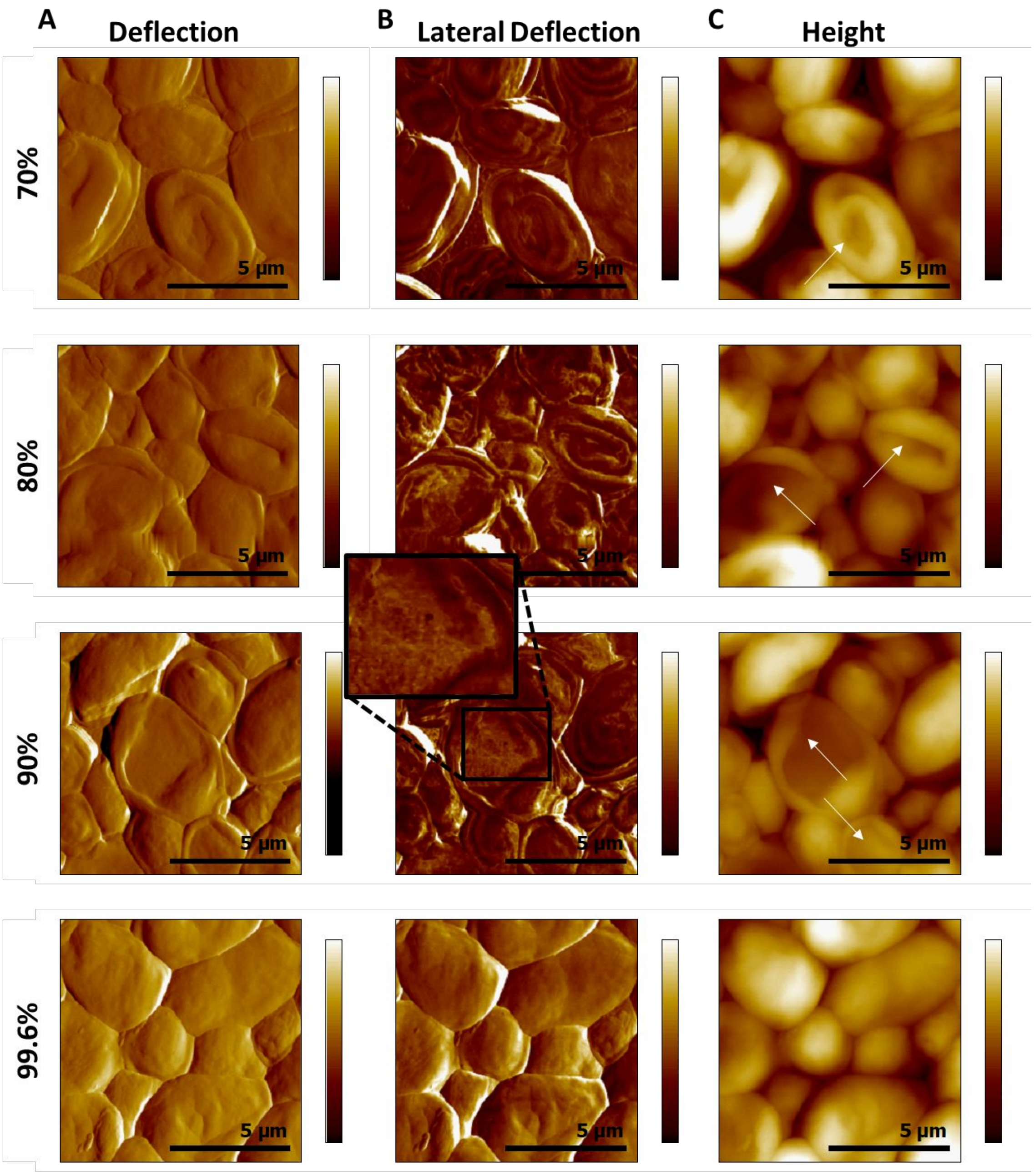

Figure S1. AFM-IR analysis of *Candida albicans* under ethanol fixation at different concentrations. Panel A: Deflection: vertical cantilever bending signal (with setpoint offset), providing enhanced edge contrast. Panel B: Lateral Deflection (torsional/sideways response) characterization: reveals frictional and lateral mechanical properties related to cell wall composition and structural heterogeneity under different ethanol fixation concentrations. Panel C: Height: cell surface topography. Visible areas of subsidence are indicated by white arrows; local damage to the cell wall surface is shown in the inset (90% ethanol). Scale: 5 µm. Ethanol concentrations: 70%, 80%, 90%, 99.6%.

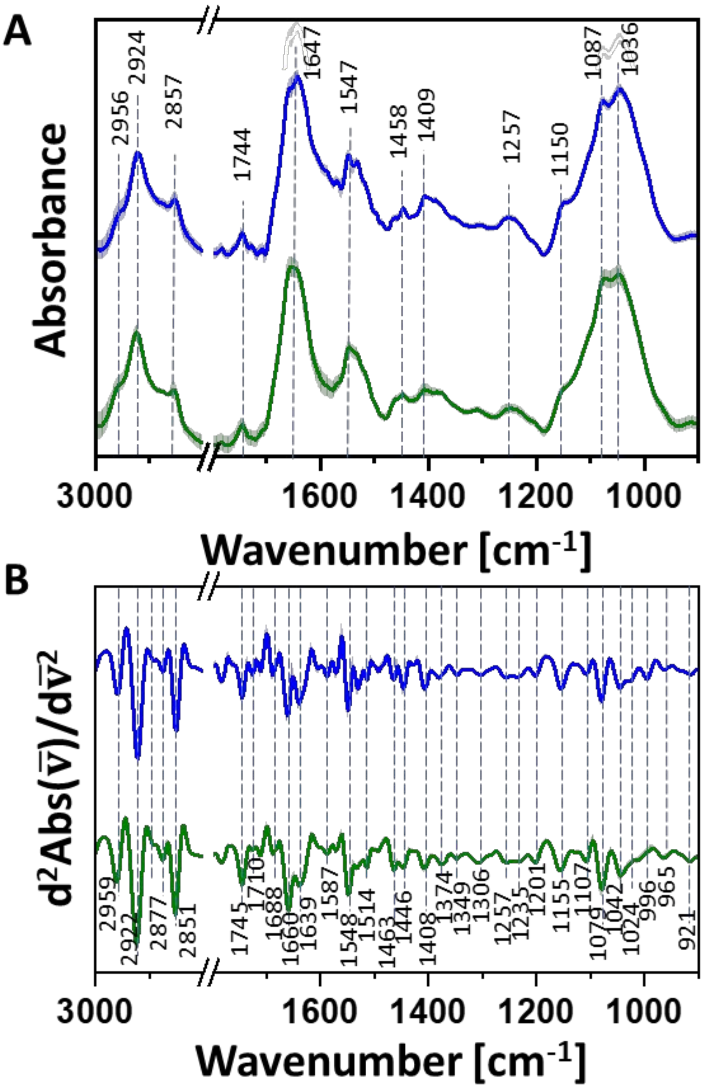

Figure S2. Global FT-IR characterization of *C. albicans* after ethanol-fixation. Panel A: Average FT-IR absorption spectra (solid lines) with standard deviations (shaded regions) for non-fixed and ethanol-fixed *C. albicans* (blue and green, respectively). Panel B: Average second-derivative analysis of the FT-IR spectra.

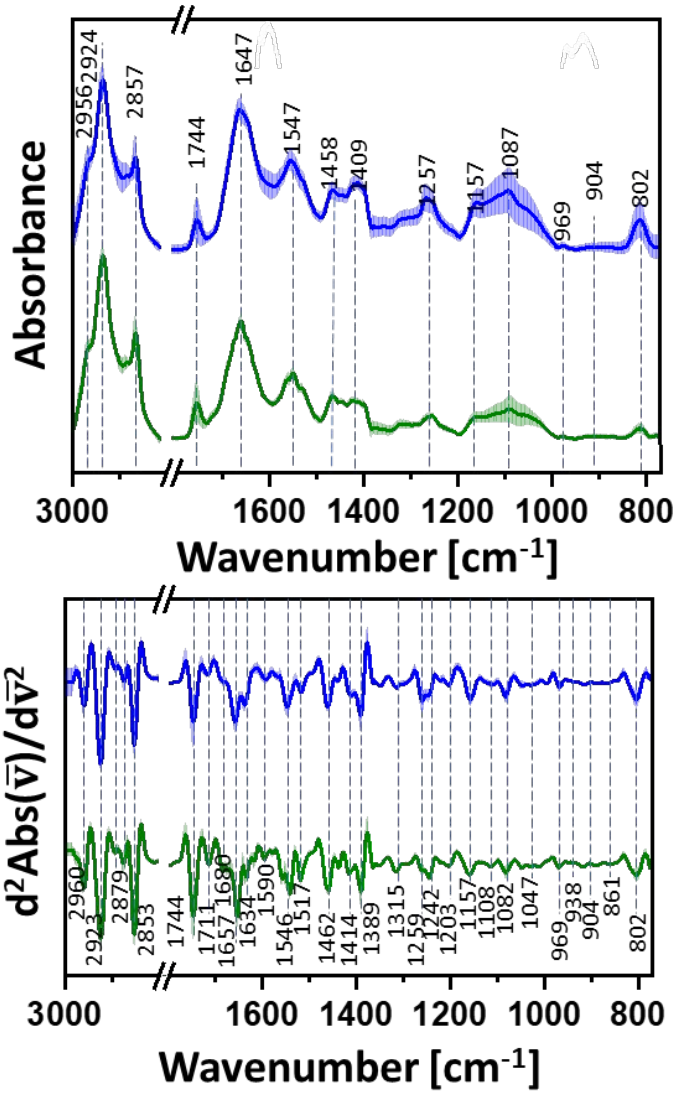

Figure S3. Nanoscale IR characterization of *C. albicans* after ethanol fixation. Panel A: Average AFM-IR absorption spectra (solid lines) with standard deviations (shaded regions) for non-fixed and ethanol-fixed *C. albicans* (blue and green, respectively). Panel B: Average second-derivative analysis of the AFM-IR spectra.

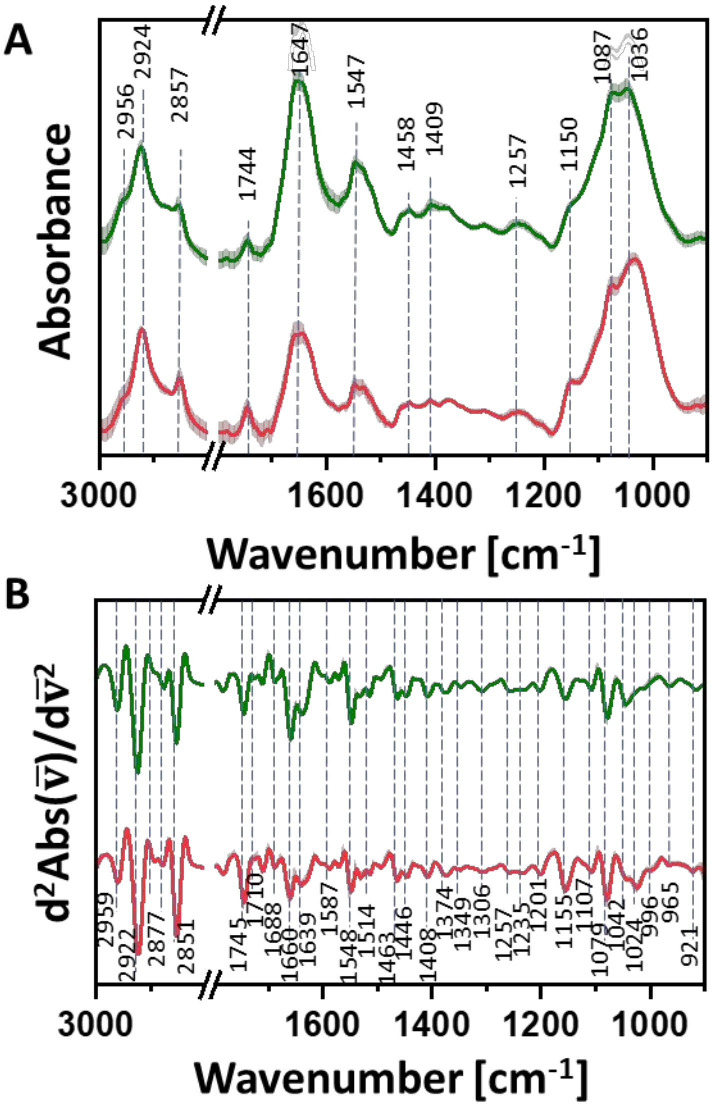

Figure S4. Global FT-IR characterization of *C. albicans* and *C. auris*. Panel A: Average FT-IR absorption spectra (solid lines) with standard deviations (shaded regions) for *C. albicans* and *C. auris* (green and red, respectively). Panel B: Average second-derivative analysis of the FT-IR spectra.

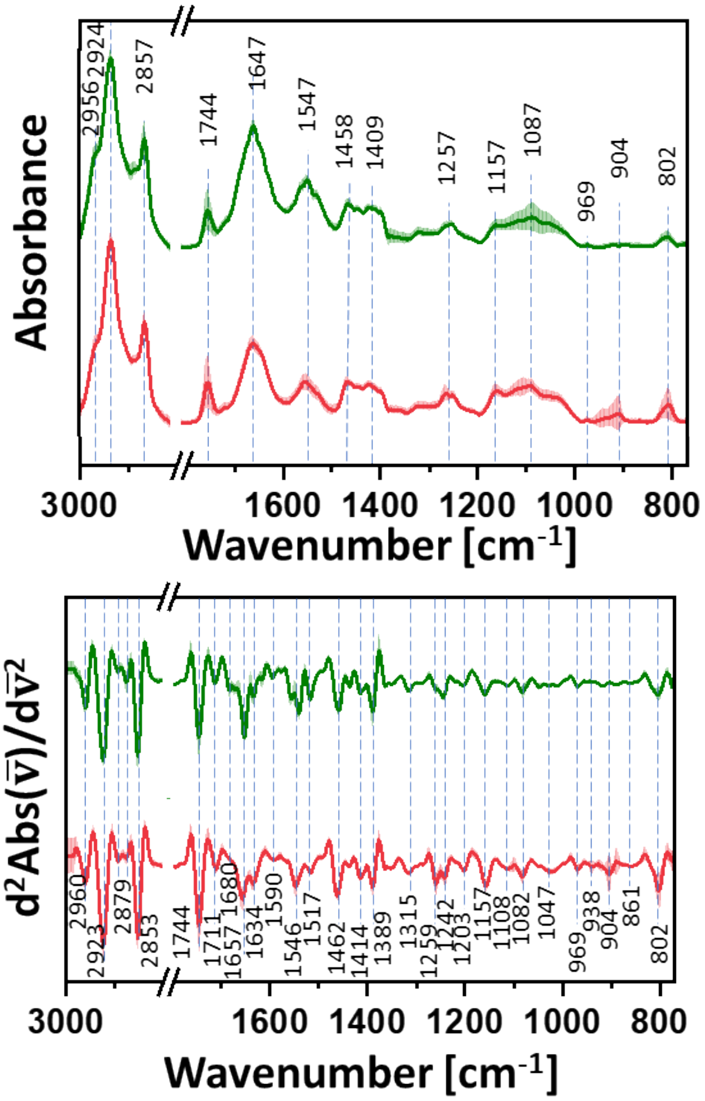

Figure S5. Nanoscale IR characterization of *C. albicans* and *C. auris*. Panel A: Average AFM-IR absorption spectra (solid lines) with standard deviations (shaded regions) for *C. albicans* and *C. auris* (green and red, respectively). Panel B: Average second-derivative analysis of the AFM-IR spectra.
